# Fucoidan Can Function as an Adjuvant In Vivo to Enhance Dendritic Cell Maturation and Function and Promote Antigen-Specific T Cell Immune Responses

**DOI:** 10.1371/journal.pone.0099396

**Published:** 2014-06-09

**Authors:** Jun-O Jin, Wei Zhang, Jiang-Yuan Du, Ka-Wing Wong, Tatsuya Oda, Qing Yu

**Affiliations:** 1 Shanghai Public Health Clinical Center, Shanghai Medical College, Fudan University, Shanghai, China; 2 Graduate School of Science and Technology, Nagasaki University, Nagasaki, Japan; 3 Department of Immunology and Infectious Diseases, The Forsyth Institute, Cambridge, Massachusetts, United States of America; Centro de Pesquisa Rene Rachou/Fundação Oswaldo Cruz (Fiocruz-Minas), Brazil

## Abstract

Fucoidan, a sulfated polysaccharide purified from brown algae, has a variety of immune-modulation effects, including promoting antigen uptake and enhancing anti-viral and anti-tumor effects. However, the effect of fucoidan *in vivo*, especially its adjuvant effect on *in vivo* anti-tumor immune responses, was not fully investigated. In this study, we investigated the effect of fucoidan on the function of spleen dendritic cells (DCs) and its adjuvant effect *in vivo*. Systemic administration of fucoidan induced up-regulation of CD40, CD80 and CD86 expression and production of IL-6, IL-12 and TNF-α in spleen cDCs. Fucoidan also promoted the generation of IFN-γ-producing Th1 and Tc1 cells in an IL-12-dependent manner. When used as an adjuvant *in vivo* with ovalbumin (OVA) antigen, fucoidan promoted OVA-specific antibody production and primed IFN-γ production in OVA-specific T cells. Moreover, fucoidan enhanced OVA-induced up-regulation of MHC class I and II on spleen cDCs and strongly prompted the proliferation of OVA-specific CD4 and CD8 T cells. Finally, OVA immunization with fucoidan as adjuvant protected mice from the challenge with B16-OVA tumor cells. Taken together, these results suggest that fucoidan can function as an adjuvant to induce Th1 immune response and CTL activation, which may be useful in tumor vaccine development.

## Introduction

Dendritic cells (DCs) are professional antigen presenting cells (APCs) and key modulators of T- and B-cell immunity mainly owing to their superior ability to take up and present antigens [1], [2]. Different subsets of DCs show differences in the abilities and modes of antigen-presentation. CD8α^+^ conventional DCs (cDCs), a minor population among total mouse spleen DCs, have the selective ability to cross-present exogenous antigens to induce cytotoxic T cell (CTL) activation [3]–[5]. In contrast, the extracellular antigens are captured and moved to endosome/lysosome in CD8α^−^ cDCs and, degraded to antigenic peptides, which are complexed with MHC class II molecules and recognized by CD4 T cells [6]. DCs can also directly sense pathogen components by pattern recognition receptors (PRRs), such as toll like receptors (TLRs), scavenger receptors (SRs), C-type lectins, mannose receptors and complement receptors [7]. The activation of these receptors induces signal events that regulate the expression of pro-inflammatory and immune mediators [7]–[9].

Tumor vaccines seek to induce CTL responses against tumors [2]. To achieve efficient tumor cell killing, different strategies have been evaluated for inducing T cell responses against tumor antigens [2], [10]. Since DC activation has critical importance for the induction of protective immune responses, induction of DC maturation was included in vaccine protocols [2], [11], [12]. However, most DC-dependent vaccine protocols have relied on *in vitro*-generated monocyte-derived DCs (MDDCs) loaded with tumor antigens [13]. Moreover, loading DCs with a tumor antigen alone is often not sufficient to stimulate effective immune responses against tumor, and inclusion of an adjuvant within a vaccine can enhance the immune activity against tumor and potentially reduce the amount of antigen required [14].

Fucoidan is a sulfated polysaccharide extracted from marine brown seaweeds and possesses certain biological activities including anti-inflammatory properties and anti-tumor effects [15], [16]. In an *in vitro* functional test, fucoidan was shown to enhance phagocytic activity of macrophages [17]. These effects promote the activation of natural killer (NK) cells, resulting in enhancement of pro-inflammatory cytokine production and anti-viral action [18]. Moreover, fucoidan can potently induce production of interferon-γ (IFN-γ) by CD4 and CD8 T cells and induce T cell cytotoxicity against antigen-expressing human cancer cells or bacteria [19], [20]. In addition, fucoidan has been shown to induce activation and maturation of human and mouse DCs *in vitro* [21]–[23]. Although many reports indicate that fucoidan exhibits various bioactivities in innate and adaptive immune cells, the effect of fucoidan on immune response *in vivo*, especially its potential effect as an adjuvant for *in vivo* anti-tumor immune responses, was not fully investigated. We hypothesize that fucoidan may function as an adjuvant and stimulate DCs to prime antigen-specific T cell responses *in vivo*, and the current study was undertaken to test this hypothesis.

## Results

### Fucoidan promotes maturation of spleen cDCs

**Figure 1:**
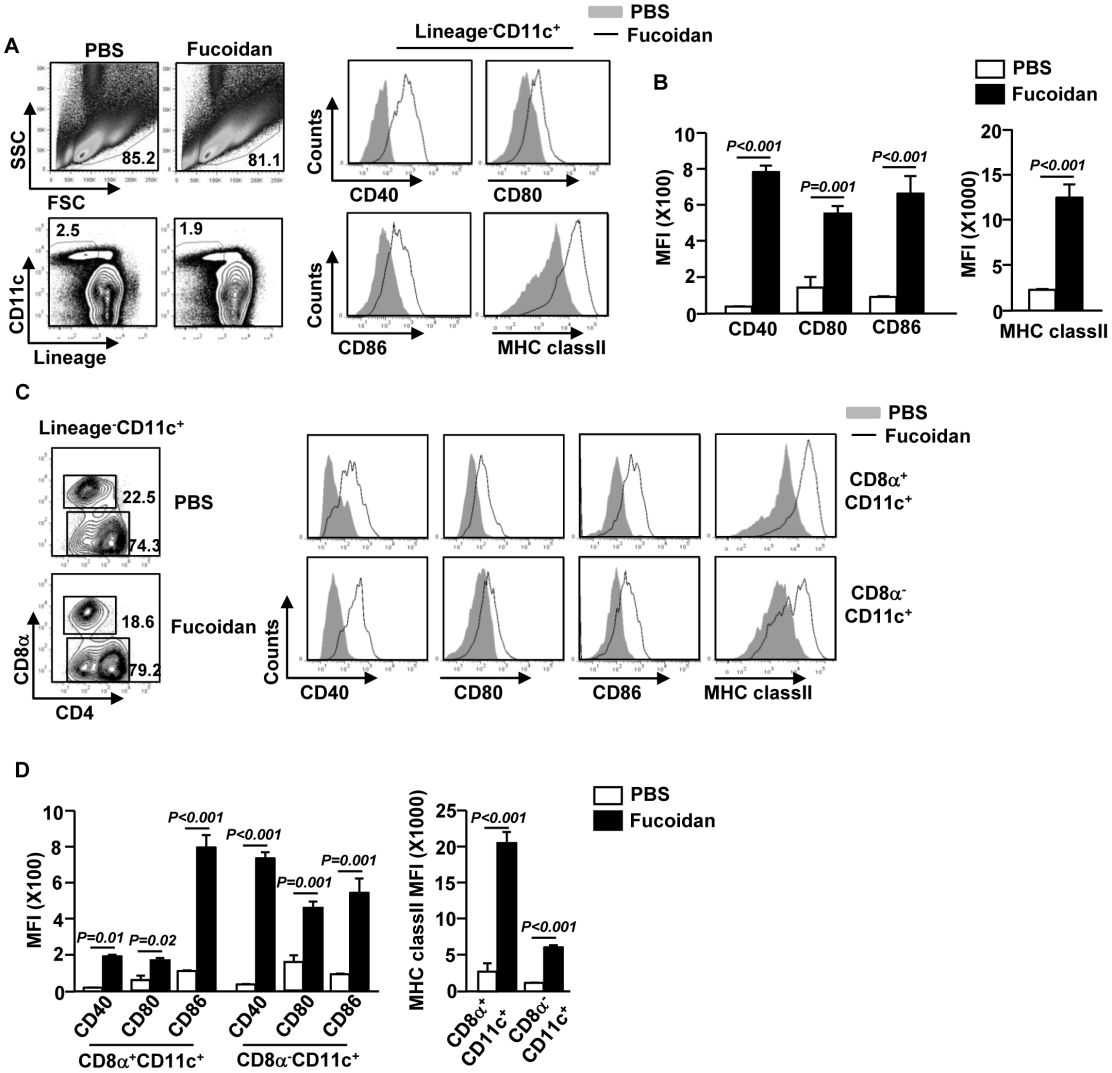
*In vivo* administration of fucoidan induces spleen cDC maturation. C57BL/6 mice were treated with 10 mg/kg fucoidan for 24 hrs. (A) Flow cytometric analysis of CD40, CD80, CD86 and MHC class II expression on the gated lineage^−^CD11c^+^ cDCs in splenocytes (upper panels). Lineage markers included CD3, Thy1.1, B220, Gr1, CD49b and TER-119. (B) Mean fluorescence intensity (MFI) of CD40, CD80, CD86 (left panel) and MHC class II (right panel) was shown. (C) Lineage^−^CD11c^+^ cDCs were further divided into CD8α^+^ and CD8α^−^ cDCs. Expression of CD40, CD80, CD86 and MHC class II was shown by histogram. (D) MFI of CD40, CD80, CD86 (right panel) and MHC class II (left panel) on CD8α^+^ and CD8α^−^ cDCs was shown. All data are representative of or the average of analyses of 6 independent samples (2 mice per experiment, total 3 independent experiments).

Previously we have showed that fucoidan can induce maturation of human peripheral blood DCs (PBDCs) [23]. Here we assessed whether fucoidan can also induce maturation of mouse DCs *in vivo*. We injected 10 mg/kg fucoidan intraperitoneally (*i.p.*) to C57BL/6 mice for 24 hrs. Fucoidan treatment led to a substantial increase in CD40, CD80, CD86 and MHC class II expression in spleen CD11c^+^ cDCs (Figure 1A and B). We next examined the effect of fucoidan on CD8α^+^ and CD8α^−^ cDC sub-populations 24 hrs after injection of fucoidan. Expression of CD40, CD80, CD86 and MHC class II was markedly increased on both CD8α^+^ and CD8α^−^ cDCs by fucoidan treatment (Figure 1C and D). These data indicate that administration of fucoidan induces spleen cDC maturation *in vivo*.

### Fucoidan induces pro-inflammatory cytokine production from spleen cDCs

**Figure 2:**
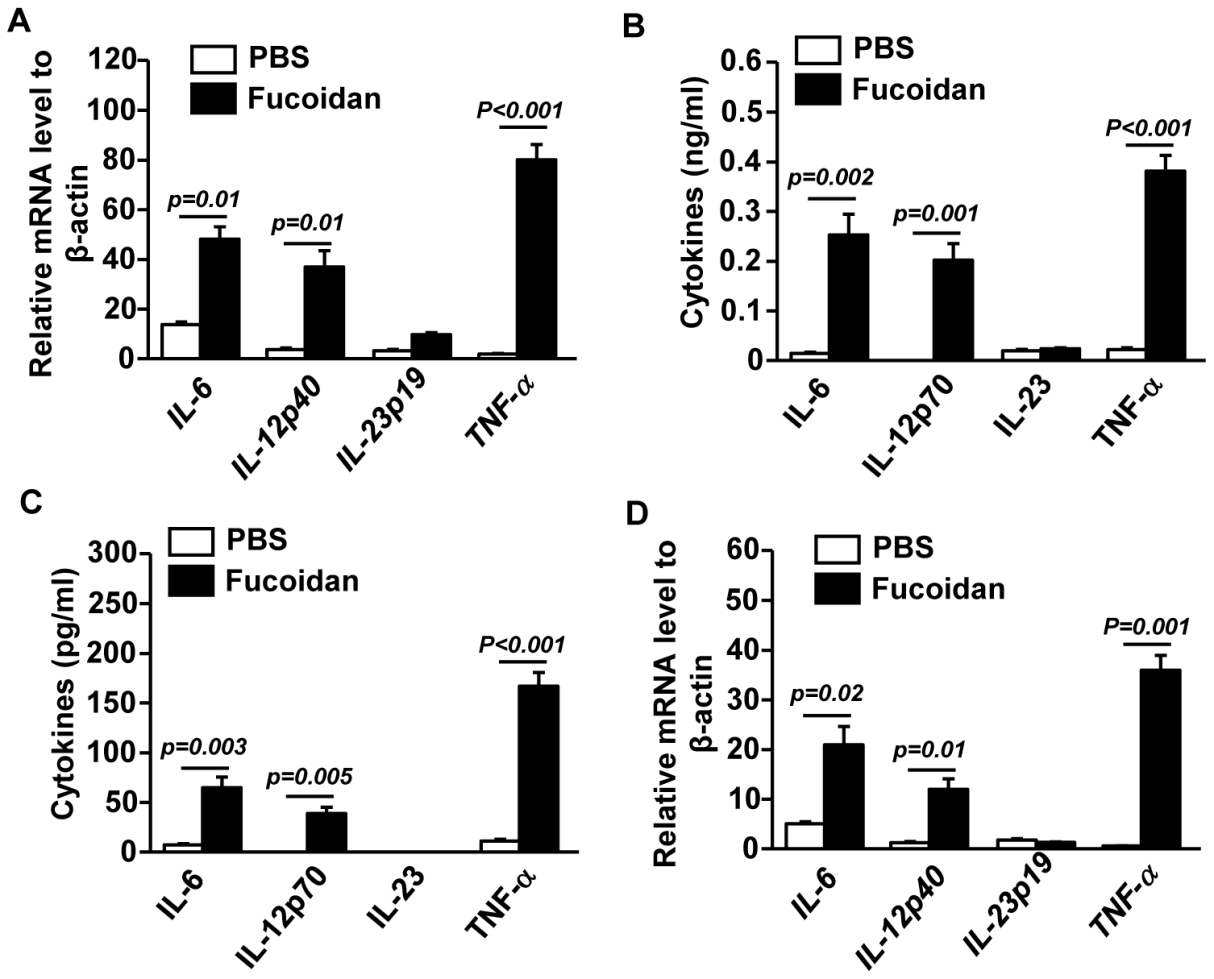
Fucoidan promotes production of pro-inflammation cytokines in cDCs. Expression levels of IL-6, IL-12p40, IL-23p19 and TNF-α mRNA in spleens were measured 3 hrs after fucoidan injection. (A) mRNA levels of IL-6, IL-12p40, IL-23p19 and TNF-α in spleens. (B) IL-6, IL-12p70, IL-23 and TNF-α concentration in serum. (C) Lineage^−^CD11c^+^ cDCs were isolated by cell sorter 2 hrs after fucoidan injection. Isolated cDCs were incubated in culture medium for 4 hrs, and then analyzed for IL-6, IL-12p70, IL-23 and TNF-α levels in the culture supernatants were measured by ELISA. (D) mRNA levels of IL-6, IL-12p40, IL-23p19 and TNF-α from isolated cDCs. All data are representative of or the average of analyses of 6 independent samples (2 mice per experiment, total 3 independent experiments).

To determine whether fucoidan affects production of cytokines, serum and spleens were collected from C57BL/6 mice 3 hrs after fucoidan administration and analyzed for pro-inflammatory cytokines. Fucoidan treatment induced up-regulation of IL-6, IL-12p40 and TNF-α mRNA levels but not IL-23p19 mRNA in splenocytes (Figure 2A). The serum levels of IL-6, IL-12p70 and TNF-α were also dramatically increased in mice treated with fucoidan (Figure 2B). Consistent with IL-23p19 mRNA levels, fucoidan did not affect serum IL-23 concentrations (Figure 2B). To specifically measure the cytokines produced by cDCs, we isolated lenease^-^CD11c^+^ cDCs from splenocytes by cell sorter 2 hrs after fucoidan administration, and then further incubated the cells in culture medium for 4 hrs Fucoidan treatment induced a marked increase in the production of IL-6, IL-12p70 and TNF-α in cultured medium (Figure 2C). Furthermore, purified CD11c^+^ cDCs from mice treated with fucoidan for 2 hrs had dramatically higher IL-6, IL-12p40 and TNF-α mRNA levels than those from control mice (Figure 2D). Therefore, systemic administration of fucoidan induced maturation of spleen cDCs as indicated by up-regulation of co-stimulatory molecules and production of pro-inflammatory cytokines.

### Fucoidan promotes generation of Th1 and Tc1 cells in an IL-12-dependent manner in vivo

**Figure 3:**
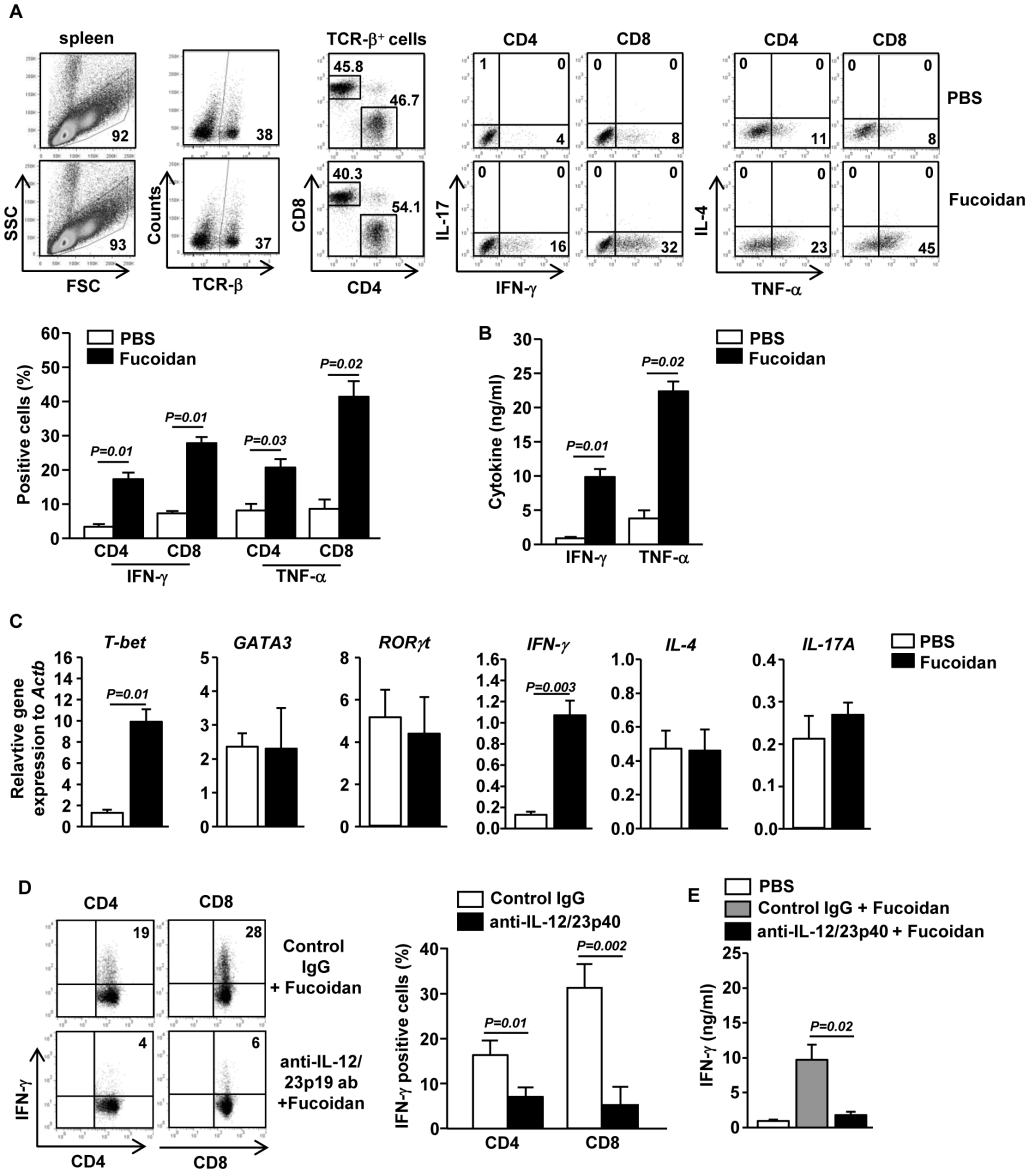
Fucoidan-induced cDC maturation promotes generation of IFN-γ-producing T cells in an IL-12 dependent manner. C57BL/6 mice were injected *i.p*. with 10 mg/kg fucoidan and 3 days later, injected again with same amount of fucoidan. (A) Percentage of IFN-γ, IL-17, IL-4 and TNF-α positive cells within CD4 and CD8 T cells in spleen was assessed by flow cytometric analysis (upper panel). Percentage of IFN-γ^+^ or TNF-α^+^ cells (lower panel). (B) IFN-γ and TNF-α levels in serum. All data are representative of or the average of analyses of 6 independent samples (2 mice per experiment, total 3 independent experiments). (C) Gene expression in spleens was measured 24 hrs after fucoidan injection. Data are the average of analyses of 6 independent samples (2 mice per experiment, total 3 independent experiments). (D) Fucoidan was injected to C57BL/6 mice in conjunction with a neutralizing anti-IL-12/23p40 antibody or control rat IgG and the same injections were repeated 3 day later. Intracellular IFN-γ expressions in CD4 or CD8 T cells from these mice were analyzed on a flow cytometer. (E) Serum IFN-γ levels from mice described in (D). All data are representative of 6 samples from 3 independent experiments.

Since fucoidan induced CD8α^+^ and CD8α^−^ cDC maturation, we assessed whether fucoidan-induced maturation of spleen cDCs can subsequently promote CD4 and CD8 T cell responses *in vivo*. Mice were *i.p*. injected with 10 mg/kg fucoidan and 3 days later, injected with the same amount of fucoidan again. Fucoidan treatment led to marked increases in the proportions of CD4 and CD8 T cells in the spleen that produced IFN-γ and TNF-α, the signature cytokines of Th1 and Tc1 cells (Figure 3A). In comparison, the percentages of IL-17- or IL-4-producing CD4 and CD8 T cells in the spleen were not increased by fucoidan (Figure 3A). Serum levels of IFN-γ and TNF-α were also markedly increased by fucoidan (Figure 3B). Moreover, fucoidan-treated mice had significantly higher amounts of T-bet (*p = 0.01*), the critical transcription factor for Th1 and Tc1 cells, and IFN-γ (*p = 0.003*) mRNA in the spleen than control mice (Figure 3C). In contrast, the mRNA levels of GATA3 and RORγt, transcription factor for Th2 and Th17, were not altered by fucoidan treatment (Figure 3C).

We next examined whether fucoidan-induced enhancement of Th1 and Tc1 responses is dependent on IL-12, a dominant inducer of Th1 and Tc1 cells in various immune responses. We injected anti-IL-12/23p40 Ab into C57/B6 mice that have received prior injection of fucoidan or PBS. The promoting effect of IFN-γ production in CD4 and D8 T cells by fucoidan administration was almost completely abrogated by IL-12/23p40 neutralization (Figure 3D). Moreover, fucoidan-induced increases in serum IFN-γ levels were also completely abrogated by anti-IL-12/23p40 treatment (Figure 3E). Hence, fucoidan promotes the generation of IFN-γ-producing Th1 and Tc1 cells in an IL-12-dependent manner. Together with the observation that fucoidan enhances IL-12 production by DCs, these data suggest that fucoidan promotes Th1 and Tc1 responses by enhancing IL-12 production.

### Fucoidan functions as an adjuvant to enhance OVA-specific antibody production and T cell responses in vivo

**Figure 4:**
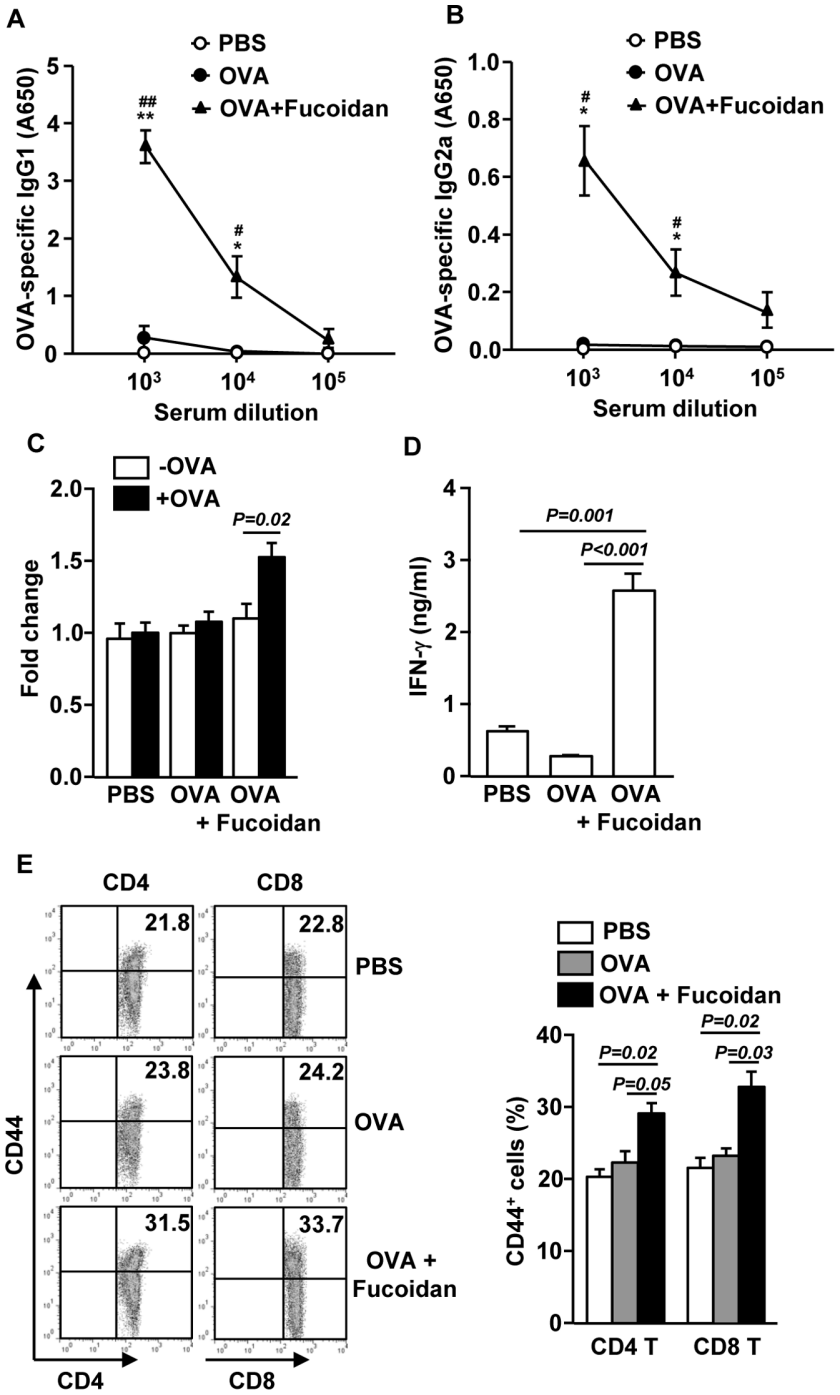
Fucoidan provides an adjuvant effect on OVA-induced B and T cell responses. C57BL/6 mice were immunized i.p with PBS, OVA or OVA + fucoidan on days 0, 15, 30. On day 35, serum OVA-specific IgG1 (A) and IgG2a (B) concentrations were measured by ELISA. *P<0.05, **P<0.01 versus OVA group. ^#^ *P<0.05*, ^##^ *P<0.01* versus PBS group. (C) Splenocytes were harvested from immunized mice on day 35, and re-stimulated with or without OVA (50 µg/ml) for 4 days. Cell proliferation from re-stimulated splenocytes was measured. (D) IFN-γ concentrations in the above splenocytes culture supernatants were shown. (E) CD44 expression on CD4 or CD8 T cells was analyzed on a flow cytometer (left panel). Percentage of CD44^+^ cells in CD4 or CD8 T cells was shown (right panel). All data are representative of 6 samples from 3 independent experiments.

To determine whether fucoidan exhibits adjuvant effect *in vivo*, we immunized mice with OVA and fucoidan, and examined specific antibody production and T cell responses against OVA. C57BL/6 mice were injected *i.p*. with OVA alone or together with 10 mg/kg fucoidan on day 0, 15 and 30. On day 35, sera were analyzed for OVA-specific IgG1 and IgG2a. Mice immunized with OVA + fucoidan produced remarkably higher amounts of anti-OVA IgG1 and IgG2a than control mice immunized with OVA alone (Figure 4A and B). On day 35, splenocytes were also harvested, re-stimulated with OVA *in vitro* for 4 days, and then analyzed for OVA-induced T cell responses. Splenocytes from mice immunized with OVA + fucoidan showed significantly greater cell proliferation and IFN-γ production than those from control mice immunized with OVA alone (Figure 4C and D). These results indicate that fucoidan could function as an adjuvant by promoting Th type immune responses. We next examined whether fucoidan promotes the generation of effector/memory T cells in OVA immunized mice based on the surface expression of CD44. As shown Figure 4E, fucoidan injection led to a marked increase in the proportions of CD44^+^ CD4 and CD8 T cells (Figure 4 E). These data suggest that fucoidan function as an adjuvant to enhance antigen specific T and B cell immune responses.

### Fucoidan adjuvant enhances antigen presentation and antigen specific T cell proliferation

**Figure 5:**
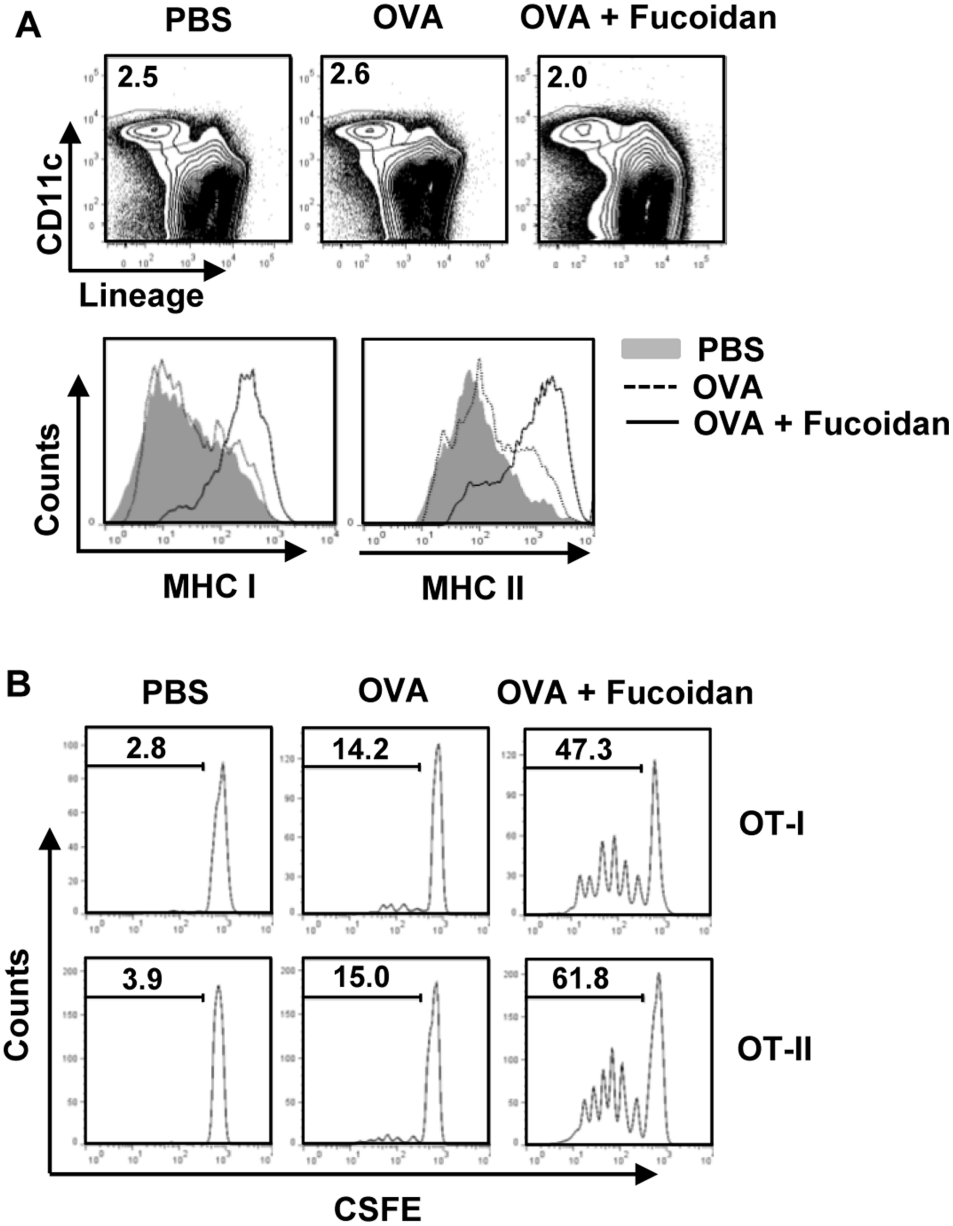
Fucoidan promotes antigen presentation and antigen-specific T cell proliferation *in vivo*. (A) C57BL/6 mice were injected with PBS, OVA or OVA + fucoidan for 24 hrs, and the expression levels of MHC class I and II on the gated Lineage^−^CD11c^+^ cDCs in splenocytes from these mice were analyzed. (B) Purified CD8 T cells from OT-I or CD4 T cells from OT-II mice were labeled with CFSE and transferred into CD45.1 congenic mice, and 24 hrs later, mice were injected with PBS, OVA or OVA + fucoidan. After 3 day treatment, splenocytes from these mice were stained for CD45.2 to identify the donor OT-I or OT-II cells and the proliferation of these cells was determined by CFSE dilution. All data are from analyses of 6 individual mice each group (2 mice per experiment, total 3 independent experiments).

To further demonstrate the adjuvant effect of fucoidan in antigen-specific T cell response *in vivo*, we first examined whether fucoidan can promote antigen-presentation or cross presentation by DCs. Mice were injected with PBS, OVA or OVA + fucoidan for 24 hrs, and then measured for expression of MHC class I and II on spleen Lineage^−^CD11c^+^ cDCs. As shown Figure 5A, spleen CD11c^+^ cDCs dramatically up-regulated surface expression of MHC class I and II molecules after treatment with OVA + fucoidan, whereas those treated with OVA alone did not. Next, we performed an adoptive transfer experiment to detect OVA specific OT-I and OT-II T cell proliferation. CFSE-labeled OT-I CD 8 T cells or OT-II CD4 T cells were transferred into CD45.1 congenic mice and 24 hrs later, the mice received injection of PBS, OVA or OVA + fucoidan. After 3 days, the proliferation of OT-I and OT-II cells was determined by CFSE dilution assay. OT-I and OT-II T cells proliferation was robustly increased in mice immunized with OVA + fucoidan compared to those in mice immunized with OVA alone (Figure 5 B). These data demonstrated that fucoidan functions as an adjuvant to enhance antigen presentation and antigen-specific CD4 and CD8 T cell activation.

### OVA-immunization with fucoidan as an adjuvant protects mice from a challenge with B16-OVA tumor cells

**Figure 6:**
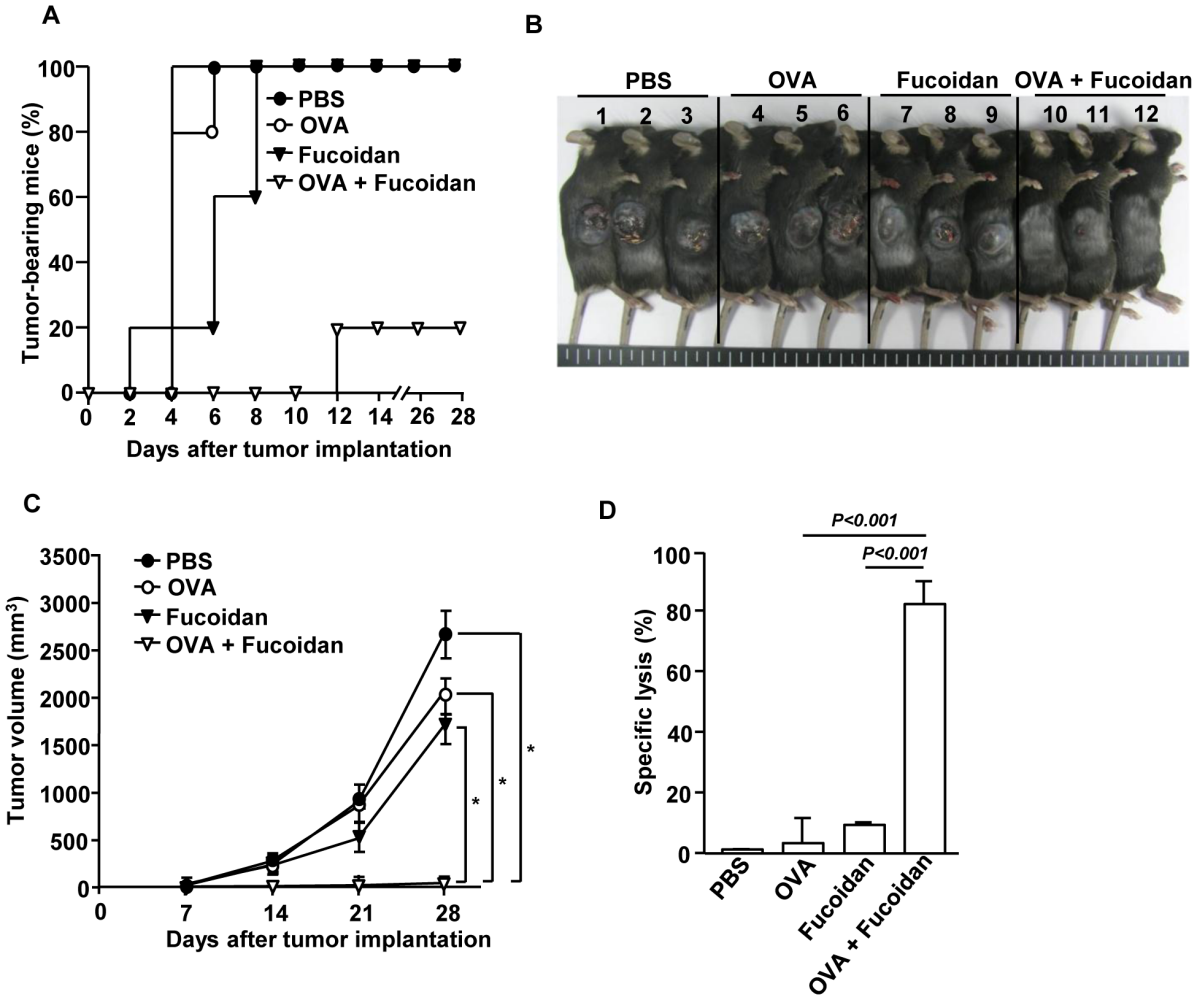
Immunization with OVA and fucoidan protects mice from challenge with B16-OVA tumor cells. C57BL/6 mice were immunized with PBS, OVA, fucoidan or OVA + fucoidan on days 0, 15 and 30. On day 35 of immunization, the mice were challenged *s.c*. with 1×10^6^ B16-OVA (melanoma) tumor cells. (A) The percentage of tumor-bearing mice and (B) the picture of tumor bearing mice are shown. (C) Tumor growth curves are shown. All data are representative of or the average of analyses of 5 independent samples (2 or 3 mice per experiment, total 2 independent experiments). *, statistically significant values, defined as *P<0.01* and determined with paired Student's t test, compared with corresponding groups. (D) On day 35, *in vivo* killing of adoptively transferred SIINFEK-coated and CFSE-labeled target cells by CTLs in the immunized mice was measured. Data are from analyses of 6 individual mice each group (2 mice per experiment, total 6 independent experiments).

Based on the observation that fucoidan functioned as an adjuvant to activate OVA-specific CD4 and CD8 T cells, we further investigated whether this response can protect mice grafted with OVA-expressing B16 tumor cells. C57BL/6 mice were immunized *i.p*. with PBS, OVA, fucoidan or OVA + fucoidan on days 0, 15 and 30 and were inoculated *s.c*. with B16-OVA tumor cells on day 35. Mice immunized with OVA + fucoidan were almost completely protected from B16-OVA tumor challenge (Figure 6A, B and C), and moreover, they did not develop tumor after a second tumor challenge, indicative of formation of long-term memory (data not shown). We also investigated the functional activity of CTL in an *in vivo* cytotoxicity assay. On day 35 after the initial immunization, the immunized mice received SIINFEKL-pulsed and CFSE-labeled splenocytes from C57BL/6 donor mice. Specific target cell lysis was 80% in mice immunized with OVA + fucoidan, indicative of T cell memory induction (Figure 6D). No significant killing was observed in mice immunized with OVA or fucoidan alone. Collectively, these data suggest that fucoidan may induce cross-presentation of OVA by DCs, resulting in the priming of OVA-specific CTLs that kill the target cells *in vivo*.

## Discussion

Fucoidan, a sulfated polysaccharide purified from brown algae, has been reported to prevent certain viral and bacterial infections [18], [25] and enhances anti-tumor activities as demonstrated by *in vitro* and *in vivo* studies [16], [19], [26], [27]. Although a variety of biological activities of fucoidan have been reported, its immune-related functions and potential adjuvant effect in the *in vivo* settings were not fully investigated. In this study, we demonstrated that *in vivo* administration of fucoidan induces maturation of spleen cDCs and activation of T cells. OVA immunization in the presence of fucoidan stimulated OVA-specific antibody production and primed OVA-specific Th1 and CTL responses, which together protected mice against the challenge of B16-OVA tumor cells. These data clearly demonstrate the adjuvant activity of fucoidan.

It has been reported that CD8α^+^CD11c^+^ cDCs can efficiently cross-present exogenous soluble and cell-bound antigens through MHC class I [3]–[5]. In contrast, CD8α^−^ cDCs present the extracellular exogenous antigens through MHC class II and direct presentation [6]. Because CD8α^+^CD11c^+^ cDCs are highly specialized in cross-priming CTL response, tumor vaccine has been designed to mainly target this DC subpopulation. In this study, we demonstrated that fucoidan administration induces maturation of both CD8α^+^CD11c^+^ and CD8α^−^CD11c^+^ cDCs *in vivo*. Moreover, systemic administration of OVA + fucoidan induced dramatic up-regulation of MHC class I and II on DCs and induced proliferation of OT-I and OT-II T cells. These data suggest that fucoidan may have the ability to enhance not only direct presentation of OVA by CD8α^−^cDCs but also cross-presentation of OVA by CD8α^+^ cDCs. Since fucoidan can induce activation of macrophages [28] and other DC populations, such as langerhans cells (LCs), that are also able to cross-prime CTLs [29], [30], we are currently investigating whether fucoidan can induce CTL responses in mice that are depleted of macrophage and LCs.

An ideal vaccine adjuvant should boost both humoral and cell-mediated immune responses in order to effectively eliminate pathogens [10], [14]. Adjuvants perform the important function of shaping the adaptive immune response, and may help immune system produce the most effective CTLs against a particular pathogen [10], [14], [31]. However, there are very few vaccine adjuvants approved for human use. Moreover, the challenge remains for developing an adjuvant that will generate Th1-polarized and antigen specific CTL responses to soluble protein antigens [10], [14], [31]. Our findings indicate that fucoidan exhibits an adjuvant activity of priming both Th1 and CTL responses to the soluble OVA antigen. We found that fucoidan positively regulates the number of IFN-γ-producing CD4 and CD8 T cells in spleen, which is associated with increased T-bet expression. To rule out the possibility that fucoidan can directly induce T cell activation and function independently of antigen presenting cells, we treated purified CD4 or CD8 T cells with fucoidan *in vitro* and found that fucoidan did not affect the generation of IFN-γ-producing CD4 or CD8 T cells (data not shown). We also showed that CD4 and CD8 T cell activation by fucoidan is dependent on IL-12 and that fucoidan can stimulate DCs to produce IL-12. Importantly, fucoidan induced up-regulation of IL-12p40 mRNA however it did not affect IL-23p19 mRNA levels. Moreover, serum levels of IL-23 were not up-regulated by fucoidan administration but IL-12p70 was up-regulated, suggesting that the promoting effect of fucoidan on Th1 and CTL responses may be achieved by enhancing IL-12 production from DCs.

Fucoidan stimulates macrophage and DC activation via scavenger receptor-A (SR-A) in *in vitro* studies [19], [23], [32], and it is likely that fucoidan may stimulate *in vivo* spleen cDCs by engaging SR-A. Activation of SR-A results in human peripheral blood DC (PBDC) maturation that subsequently promotes Th1 responses [23]. DCs are known to prime CTL responses upon activation by ligands targeting several PRRs, including toll-like receptors and Dectin-1 [33], [34]. Thus, it may be likely that stimulation of SR-A on DCs by fucoidan results in the cross-priming of OVA-specific CTLs. Similar to our *in vivo* observations, fucoidan has been shown to enhance CTL activity against NY-ESO-1 expressing human cancer cells *in vitro* [19]. Our future studies will directly test whether fucoidan can activate SR-A and whether activation of SR-A signaling in DCs can promote CLR responses *in vivo* by using SR-A-knock out mouse.

In conclusion, our results provide evidence that the fucoidan produced by *Fucus vesiculosus* is a novel adjuvant, which can stimulate DC maturation, CTL activation, Th1 immune responses, antigen specific antibody production and memory T cell generation. The adjuvant function of fucoidan will be potentially useful for tumor vaccines.

## Materials and Methods

### Mice and cell lines

C57BL/6 mice (6 weeks old) were purchased from the B&K Laboratory Animal Corp (Shanghai). OT-I and OT-II TCR transgenic mice and C57BL/6-Ly5.1 (CD45.1) congenic mice were obtained from Shanghai Public Health Clinical Center, and kept under pathogen-free conditions. All experiments were carried out under the guidelines of the Institutional Animal Care and Use committee at the Shanghai Public Health Clinical Center. The protocol was approved by the committee on the Ethics of Animal Experiments of the Shanghai Public Health Clinical Center (Mouse Protocol Number: SYXK-2010-0098). The murine melanoma cell line B16F10 (ATCC, CRL-6475) expressing OVA (B16-OVA) was cultured in 10% FCS RPMI (Sigma Aldrich, 2 mM glutamine, 1 M HEPES, 100 µg/ml streptomycin and 100 U/ml penicillin, 2 mM 2-mercaptoethanol). All cell lines were cultured at 37°C in a humidified atmosphere of 5% CO_2_ and air.

### Chemicals and cytokines

Fucoidan of *Fucus vesiculosus* and chicken ovalbumin (OVA) were obtained from Sigma-Aldrich. The endotoxin levels in commercially obtained fucoidan were evaluated using a Limulus amebocyte lysate (LAL) assay kit (Lonza). Fucoidan and OVA used in all experiments contained less than 0.1 endotoxin unit/ml. The H2-K^b^ restricted peptide OVA257–264 (SIINFEKL) was purchased from Chinapeptides (China).

### Antibodies

Isotype control antibodies (Abs) (IgG1, IgG2a or IgG2b), CD11c (HL3), CD4 (GK1.5), CD8α (YTS169.4), CD40 (3/23), CD80 (16-10A1), CD86 (GL-1), anti-IL-4 (11B11) and anti-IL-12/23p40 (C17.8) were from BioLegend; anti-MHC class I (AF6-88.5.3), anti-MHC class II (M5/114.15.2), anti-IFN-γ (XMG1.2), anti-IL-17 (TCC11-18H10.1) and anti-TNF-α (MP6-XT22) were from eBioscience.

### Flow cytometry analysis

Cells were washed with phosphate buffered saline (PBS) containing 0.5% BSA, pre-incubated for 15 min with unlabeled isotype control Abs, and then labeled with fluorescence-conjugated Abs by incubation on ice for 30 min followed by washing with PBS. Cells were analyzed on a FACS Aria II (Becton Dickinson) and FlowJo 8.6 software (Tree Star). Cellular debris was excluded from the analysis by forward- and side-scatter gating. Dead cells were further excluded by 7 aminoactinomycin D (7AAD) (BioLegend) staining and gating on the 7AAD-negative population. As a control for nonspecific staining, isotype-matched irrelevant mAbs were used.

### Spleen DC analysis

Spleens were cut into small fragments and digested, with 2% fetal bovine serum (FCS) containing collagenase for 20 min at room temperature. Cells from the digest were centrifuged and the cell pellet was resuspended in 5 mL of 1077 histopaque (Sigma-Aldrich). More histopaque was then layered below the cell suspension, with EDTA-FCS-layered above it. After centrifugation at 1700 g for 10 min, the light density fraction (<1.077 g/cm3) was collected and incubated for 30 min with the following FITC-conjugated monoclonal antibodies (mAbs): anti-CD3 (17A2), anti-Thy1.1 (OX-7), anti-B220 (RA3-6B2), anti-Gr1 (RB68C5), anti-CD49b (DX5) and anti-TER-119 (TER-119). Cells were analyzed on a FACS Aria II (Becton Dickinson). The cDCs were identified as lineage^−^CD11c^+^ cells, which were further subdivided into CD8α^+^ and CD8α^−^ cDCs.

### *Ex vivo* T cell stimulation and intracellular cytokine staining

Singles cells prepared from spleens were stimulated *in vitro* for 4 hrs with phorbol 12-myristate 13-acetate (50 ng/ml) and ionomycin (1 µM; both from Calbiochem), with the addition of monensin solution (Biolegend) during the final 2 hrs. Cells were then stained for surface markers. For intracellular cytokine staining, cells were stained for surface molecules first, then fixed and permeabilized with Cytofix/Cytoperm buffer (eBioscience) and subsequently incubated with anti-cytokine antibodies in Perm/Wash buffer (eBioscience) for 30 min. Control staining with isotype control IgGs was performed in all experiments.

### ELISA

IL-6, IL-12p70, IL-23 (p19/p40) and TNF-α concentrations in the sera were measured in triplicate using standard ELISA kits (Biolegend).

### Real-time PCR

Total RNA was reverse-transcribed into cDNA using Oligo (dT) and M-MLV reverse transcriptase (Promega). The cDNA was subjected to real-time PCR amplification (Qiagen) for 40 cycles with annealing and extension temperature at 60°C, on a LightCycler 480 Real-Time PCR System (Roche). Primer sequences are: mouse β-Actin forward, 5′-TGGATGACGATATCGCTGCG-3′; reverse, 5′-AGGGTCAGGATACCTCTCTT-3′, IL-6 forward, 5′-AACGATGATGCACTTGCAGA-3′; reverse, 5′-GAGCATTGGAAATTGGGGTA-3′, IL-12p40 forward, 5′-CACATCTGCTGCTCCACAAG-3′; reverse, 5′- CCGTCCGGAGTAATTTGGTG-3′, IL-23p19 forward, 5′-CTC TCG GAATCTCTGCAT GC-3′; reverse, 5′-ACCATCTTCACACTGGATACG-3′, TNF-α forward, 5′-CCTTTCACTCACTGGCCCAA-3′; reverse, 5′-AGTGCCTCTTCTGCCAGTTC-3′ T-bet forward, 5′-CAACAACCCCTTTGCCAAAG-3′; reverse, 5′-TCCCCCAAGCATTGACAGT-3′, GATA3 forward, 5′-CGGGTTCGGATGTAAGTCGAGG-3′; reverse, 5′- GATGTCCCTGCTCTCCTTGCTG-3′, RORγt forward, 5′-CCGCTGAGAGGGCTTCAC-3′; reverse 5′-TGCAGGAGTAGGCCACATTACA-3′, IFN-γ forward, 5′-GGATGCATTCATGAGTATTGC-3′; reverse, 5′-CTTTTCCGCTTCCTGAGG-3′, IL-4 forward, 5′-ACAGGAGAAGGGACGCCAT-3′; reverse 5′-GAAGCCCTACAGACGAGCTCA-3′, IL-17A forward, 5′-GCGCAAAAGTGAGCTCCAGA-3′; reverse 5′-ACAGAGGGATATCTATCAGGG-3′.

### Mouse immunization

C57BL/6 mice were immunized *i.p*. with PBS alone, 50 µg of OVA in PBS or OVA mixed with 10 mg/kg fucoidan in PBS on days 0, 15 and 30. On day 35, mice were sacrificed, sera were collected, and splenocytes were harvested for further analysis.

### OVA-specific antibody analysis

96-well plates were coated with OVA (10 µg/ml) and blocked with 1% bovine serum albumin (BSA). Serum samples were diluted and added to each well, followed by incubation with biotin-conjugated anti-mouse IgG1 and IgG2a (Biolegend) and streptavidin-conjugated HRP. The reaction was developed by TMB substrate (Sigma), and A_650_ was measured using a plate reader.

### OT-I and OT-II T cell proliferation

CD4 T cells from OT-II mice or CD8 T cells from OT-I mice were isolated from spleens using CD4 T cell or CD8 T cell isolation kit (Miltenyi Biotec), respectively. The cells were suspended in PBS/0.1% BSA containing 10 µM CFSE (Invitrogen) for 10 min. CFSE-labeled cells (1×10^6^) were *i.v*. transferred into CD45.1 congenic mice, and 24 h later, mice were injected with PBS alone, 50 µg of OVA in PBS or OVA plus fucoidan (10 mg/kg) in PBS. At 72 h after immunization, splenocytes were harvested and OT-I or OT-II T cell proliferation was determined by analyzing the CFSE fluorescence intensity through flow cytometry.

### *In vivo* cytotoxicity assay

Mice were injected *i.v*. with a mixture of splenocytes differentially labeled with CFSE (2, 20, or 200 nM) and loaded with 1, 10, or 100 nM SIINFEKL peptide, respectively, and spleen cells labeled with 10 mM CellTracker™ Orange CMTMR (Life technologies) and not loaded with peptide. A total of 10×106 cells per mouse were injected, consisting of a mixture containing each target cell population. Splenocytes were collected 24 hr after injection of target cells. Presence of viable target cells was determined using exclusion by 7-aminoactinomycin D. Percentage killing was calculated using the formula as described [24].

### Statistical analysis

Results are expressed as the mean ± standard error of the mean. Statistical significance was determined by Student's *t*-test (two-tailed, two-sample equal variance). P values smaller than 0.05 were considered as statistically significant.
